# The most influential papers in mitral valve surgery; a bibliometric analysis

**DOI:** 10.1186/s13019-020-01214-y

**Published:** 2020-07-20

**Authors:** N. Allen, K. O’Sullivan, J. M. Jones

**Affiliations:** Department of Cardiothoracic Surgery, Royal Victoria Hospital, 274 Grosvenor Road, Belfast, BT12 6BA UK

**Keywords:** Mitral valve, Mitral valve replacement, Mitral repair, Bibliometric analysis, Valve surgery, Cardiac surgery, Cardiovascular, Minimally invasive, Percutaneous surgery, Robotic surgery

## Abstract

This study is an analysis of the 100 most cited articles in mitral valve surgery. A bibliometric analysis is a tool to evaluate research performance in a given field. It uses the number of times a publication is cited by others as a proxy marker of its impact. The most cited paper Carpentier et al. discusses mitral valve repair in terms of restoring the geometry of the entire valve rather than simply narrowing the annulus (Carpentier, J Thorac Cardiovasc Surg 86:23–37, 1983). The first successful mitral valve repair was performed by Elliot Cutler at Brigham and Women’s Hospital in 1923 (Cohn et al., Ann Cardiothorac Surg 4:315, 2015). More recently percutaneous and minimally invasive techniques that were originally designed as an option for high risk patients are being trialled in other patient groups (Hajar, Heart Views 19:160–3, 2018). Comparison of percutaneous method with open repair represents an expanding area of research (Hajar, Heart Views 19:160–3, 2018). This study will analyse the top 100 cited papers relevant to mitral valve surgery, identifying the most influential papers that guide current management, the institutions that produce them and the authors involved.

## Introduction

The first successful mitral valve surgery was performed in 1923 by Elliot C. Cutler, a student of Harvey Cushing, at Peter Bent Brigham Hospital (now incorporated in The Brigham and Women’s Hospital) [1, 2]. A blind commissurotomy via the apex of the heart was performed for a young female patient with rheumatic mitral valve stenosis. Despite being comatose from low cardiac output pre-procedure she was discharged 4 days later having made a good recovery [2]. Following this initial success others began to develop techniques to treat mitral valve disease. Sir Henry S. Souttar performed the first finger fracture of mitral stenosis in 1925. By 1964 Harken and Ellis produced a series of 1571 closed mitral valvuloplasties [3]. In 1986 Hansen et al. published a paper of trials in canine models emphasising the importance of preserving the mitral apparatus in order to maintain left ventricular function [4]. This led to a move towards repair rather than replacement that persists to the present day. More recently minimally invasive surgery, percutaneous intervention and robotic surgery have all developed into expanding areas of research [5]. To the best of our knowledge this study is the first to analyse the top cited papers in mitral valve surgery, using the number of citations as a proxy for the impact that paper has on the field. A number of other bibliometric analyses in other surgical specialties, and more broadly in cardiovascular surgical topics, have been published in recent years [6–9]. They have contributed to better understanding of the influential papers in their respective fields.

“A citation is an alphanumeric expression which acknowledges the relevance given by the author to the work of others on a topic of discussion in which the citation appears. It is an act of intellectual honesty” [6]. Using the number of citations a paper receives since its publication – citation analysis, the influence of a paper can be measured. Using a citation analysis, the impact factor of a journal can be determined. The impact factor is a measure of how many citations the average article in the journal receives in a year. These methods are not without their flaws – the intrinsic value of a paper is not directly correlated to the number of citations it receives [10]. The impact factor of a journal is determined by the papers a small number of reviewers deem suitable to be published in their journal; an inherently subjective process. While the use of citation analysis and impact factor are not perfect as measures of merit they are accepted as the best proxy measures of the impact of a paper and the journal it is published in [10]. The purpose of this paper is rank the top 100 cited papers in mitral valve surgery and analyse the journals, country of origin and top publishing institutions and authors using number of citations and impact factor as measures of impact on mitral valve surgery.

## Methods

Journals related to cardiothoracic surgery or cardiovascular themes were included based on their 2018 impact factor. We used the Clarivate Analytics Journal Citation Reports database to identify 20 Journals with high impact factor (1.062–70.67). We then used Clarivate Analytics Web of Science to search for publications with the word “mitral” as a topic in the categories of “Surgery” and “Cardiac Cardiovascular Systems”. This yielded 94,733 results of which the top cited 1250 were chosen for further review. From this list the Top 100 cited papers specific to mitral valve Surgery were selected. Published guidelines, papers on radiological investigation of mitral valve disease and purely medical management of mitral valve disease were excluded.

Once the top 100 articles were identified the title, authors, journal, country of origin, year of publication and number of citations were recorded. This data was then analysed to produce tables for the top 100 papers, the journals with the highest impact factor, the number of top 100 articles published per decade, number of articles per top journal, common research themes, authors with multiple publications, the country with the most top 100 papers and the institutions with most top 100 papers.

## Results

Eleven Journals related to cardiac surgery or mitral valve disease with a high impact factor (≥1.062) were included in the initial search (Table 1). The top 100 publications in each of these 11 journals was established (Table 2). The most frequently represented journals were *Circulation* (36 papers), *Journal of Thoracic and Cardiovascular Surgery* (34 papers) *and Annals of Thoracic Surgery* (23 papers) (Table 3). The paper with the highest number of citations (1348) was “The French Correction” by Carpentier et al. [11]. A functional classification was described with related mitral valve repair methods using extensive leaflet resection, chordal manipulation, and annuloplasty to restore a functioning valve [107]. The lowest cited paper included received 148 citations. The mean number of citations a paper received was 248. The oldest paper was published in 1960 and was ranked 64th. It describes techniques for mitral repair due to chordae tendineae rupture. Most of the papers in the top 100 were published in the 1990s (35 papers) (Table 4).

**Table 1:** List of journals included ranked by impact factor

Rank	Journal	2018 Impact factor
1	New England Journal of Medicine	70.67
2	Lancet	59.102
3	Journal of The American Medical Association	51.273
4	Circulation	23.054
5	Chest	9.657
6	Journal of Thoracic and Cardiovascular Surgery	5.261
7	Annals of Thoracic Surgery	3.919
8	European Journal of Cardio-Thoracic Surgery	3.847
9	Journal of cardiac surgery	1.179
10	Annals of thoracic and cardiovascular surgery	1.109
11	Journal of Cardiovascular Surgery	1.062

**Table 2:** List of most cited papers by first author and number of citations

Rank	First Author	Total Citations
1	Carpentier, A [11]	1348
2	Inoue, K [12]	842
3	Cannegieter, S [13]	657
4	O’Brien, S [14]	601
5	Enriquezsarano, M [15]	499
6	Carpentier, A [16]	495
7	Alfieri, O [17]	462
8	Bolling, S [18]	443
9	McGee, E [19]	414
10	Shroyer, A [20]	407
11	Carpentier, A [21]	403
12	Gillinov, A [22]	375
13	Gillinov, A [23]	374
14	Schoen, F [24]	362
15	Webb, J [25]	358
16	Carpentier, A [21]	332
17	Devereux, R [26]	330
18	Palacios, I [27]	315
19	Pibarot, P [28]	312
20	Rosenhek, R [29]	301
21	Deloche, A [30]	290
22	Bolling, S [31]	261
23	Tribouilloy, C [32]	259
24	Mohr, F [33]	253
25	Bjork, V [34]	252
26	Suri, R [35]	242
27	Carpentier, A [36]	237
28	Modi, P [37]	234
29	Edwards, F [38]	231
30	Palacios, I [39]	231
31	Maisano, F [40]	231
32	David, T	231
33	Carabello, B [41]	229
34	David, T [42]	228
35	Mohr, F	225
36	Schuler, G [43]	223
37	Cosgrove, D [44]	221
38	Lillehei, C [45]	221
39	Grossi, E [46]	218
40	Geha, A [47]	217
41	Braunberger, E [48]	214
42	Edmunds, L [49]	212
43	Edmunds, L [50]	212
44	Navia, J [51]	210
45	Shinoka, T [52]	209
46	Schoen, F [53]	208
47	Rankin, J [54]	202
48	Kron, I [55]	201
49	Rieder, E [56]	201
50	Messas, E [57]	200
51	Thomas, J [58]	199
52	Altman, R [59]	199
53	Nobuyoshi, M [60]	196
54	Schofer, J [61]	193
55	Calafiore, A [62]	191
56	Amoury, R [63]	185
57	David, T [64]	184
58	Crawford, M [65]	183
59	Fucci, C [66]	180
60	Gammie, J [67]	179
61	Wendel, H [68]	178
62	Flameng, W [69]	177
63	Rozich, J [70]	175
64	Jamieson, W [71]	174
65	Webb, J [72]	174
66	McGoon, D	174
67	Ling, L [73]	173
68	Vyavahare, N [74]	172
69	Perier, P [75]	171
70	Ambler, G [76]	171
71	Chua, Y [77]	170
72	Bolling, S [78]	169
73	Masur, H [79]	169
74	Braun, J [80]	167
75	John, S [81]	167
76	Gammie, J [82]	167
77	Bodner, J [83]	167
78	David, T [84]	163
79	Cox, J [85]	162
80	Hickey, M [86]	161
81	Kang, D [87]	160
82	Acar, J [88]	159
83	Fattouch, K [89]	159
84	King, R [90]	158
85	Abascal, V [91]	156
86	Magne, J [92]	156
87	Rossiter, S [93]	156
88	Jamieson, W [94]	155
89	Kosakai, Y [95]	153
90	Borger, M [96]	152
91	Mohty, D [97]	152
92	David, T [98]	152
93	Cohn, L [99]	151
94	Cohn, L [100]	151
95	Dreyfus, G [101]	151
96	Braun, J [102]	150
97	David, T [103]	150
98	Ben F [104]	149
99	Chauvaud, S [105]	148
100	Seeburger, J [106]	147

**Table 3:** List of journals where the most cited papers can be found

Rank	Journal	Number of Articles
1	Circulation	36
2	Journal of Thoracic and Cardiovascular Surgery	34
3	Annals of Thoracic Surgery	23
4	European Journal of Cardio-Thoracic Surgery	7

**Table 4:** Number of top cited papers per decade

Decade	Number of Articles
1960s	4
1970s	6
1980s	18
1990s	35
2000s	34
2010s	3

The topics covered followed several recurring themes. Most common were papers comparing various methods of surgical repair. Other common themes were risk stratification, rates of complication, long term outcomes and minimally invasive, percutaneous and robotic techniques (Table 5). Various first authors have multiple papers in the top 100. Tirone E. David features 6 times as first author in the top 100 with papers describing repair and replacement techniques. Alain Carpentier is listed as first author for 5 papers – the top cited papers and several others describing mitral valve repair. A further 11 authors have multiple first authorships Bolling, Braun, Cohn, Edmunds, Gammie, Gillinov, Jamieson, Mohr, Palacios, Schoen and Webb (Table 6).

**Table 5:** List of common themes from the top papers

Rank	Topic	Number of Articles
1	Repair/replacement techniques	32
2	Risk Stratification	21
3	Complications	14
4	Long term outcomes	12
5	Minimally invasive/Robotic	10
6	Percutaneous techniques	9
7	Other	2

**Table 6:** Authors with multiple first authorship of 100 most cited papers

Rank	First Author	Frequency of First Authorship
1	David, T	6
2	Carpentier, A	5
3	Bolling, S	3
4	Braun, J	2
5	Cohn, L	2
6	Edmunds, L	2
7	Gammie, J	2
8	Gillinov, A	2
9	Jamieson, W	2
10	Mohr, F	2
11	Palacios, I	2
12	Schoen, F	2
13	Webb, J	2

Looking at the country of origin; most papers were from the United States of America (50 papers) with Canada and France representing 2nd and 3rd places with 13 and 9 papers respectively. A total of 15 countries were represented individually (Table 7). The highest publishing institution was The Mayo Clinic with Toronto General Hospital and The Cleveland Clinic in second and third places (Table 8).

**Table 7:** Countries with multiple published papers

Rank	Country	Number of top 100 papers
1	USA	50
2	Canada	13
3	France	9
4	Germany	7
5	Italy	5
6	Netherlands	3
6	Japan	3
6	Austria	3
7	UK	1
7	Tunisia	1
7	South Korea	1
7	India	1
7	Finland	1
7	Belgium	1
7	Argentina	1

**Table 8:** Institutes that have published multiple most cited papers

Institution	Frequency
Mayo Clinic, Rochester, MN, USA	7
Toronto General Hospital and the University of Toronto, Toronto, ON, Canada	7
The University of Michigan, Ann Arbor, MI, USA	5
Cleveland Clinic Foundation, Cleveland, OH, USA.	5
Massachusetts General Hospital, Boston, MA, USA	5
Hôpital Européen Georges Pompidou, Université René Descartes, Paris, France.	4
Brigham and Women’s Hospital, Boston, MA, USA	4
St. Paul’s Hospital, University of British Columbia, Vancouver, BC, Canada	4
Hospital Broussais, Paris, France.	4
University Hospital Leiden, The Netherlands	3
Heart Center, University of Leipzig, Leipzig, Germany	3
Laval Hospital Research Center/Québec Heart Institute, Laval University, Québec, Canada	2
Medical University of Vienna, Vienna, Austria.	2
East Carolina Heart Institute, Greenville, NC, USA.	2
IRCCS S. Raffaele Hospital, Milano, Italy.	2
Duke Clinical Research Institute, Durham, NC, USA.	2
University of Pennsylvania School of Medicine, Philadelphia, PA, USA	2
Children’s Hospital, Boston, MA, USA.	2
University of Maryland Medical Center, Baltimore, MD, USA	2

## Discussion

The first surgical intervention on the mitral valve was performed as a blind commissurotomy for a patient in extremis by E.C. Cutler in 1923 [2]. The patient had a rheumatic valve, a common aetiology that drove early interest in mitral valve surgery. Open surgery for mitral disease did not become possible until the invention of cardiopulmonary bypass by Dr. John Gibbon in the late 1930s [108]. C. Walton Lillehei is credited with the first mitral valve repair for mitral insufficiency in 1960 [109]. The importance of preserving mitral apparatus was described by Hansen et al. leading to a preference for repair of the patient’s own valve rather than replacement wherever possible [4].

More recently mitral surgery research has begun to examine the use of percutaneous, minimally invasive and robotic surgery [110, 111]. Repair of the mitral valve has been propelled by research into new techniques for repair, comparing repair over replacement, and more recently the comparison of minimally invasive and robotic techniques compared to open repair. A number of key papers include McGoon et al. “Repair of mitral insufficiency due to ruptured chordae tendineae” [112] in 1960 and later Carpentier et al*.* “The French Correction” [11] in 1983. In 1994 Cohn et al. demonstrated the benefits of repair over replacement of the mitral valve whenever possible [113].

The most frequently cited paper in our analysis is “The French Correction” first presented by Carpentier as the Honoured Guest’s Address to The American Association for Thoracic Surgery in Atlanta 1983, emphasised the need to focus on function rather than valve lesions. This functional analysis has become the foundation of valve assessment and reconstruction.

Papers discussing percutaneous, minimally invasive and robotic techniques include “Clinical Applications of Transvenous Mitral Commissurotomy by a New Balloon Catheter” by Inoue et al. *1984*. This highly cited paper (842 citations) discussed 6 patients who had successful treatment of mitral stenosis using mitral commissurotomy using a balloon catheter inserted via the Saphenous vein.

“Transcatheter valve-in-valve implantation for failed bioprosthetic heart valves.” Webb et al. *2010* demonstrated that transcatheter valve-in-valve insertion was feasible. Their study included 24 patients, 6 of whom had their mitral valve replaced using this percutaneous method.

Regarding robotic surgery “Computer-enhanced robotic cardiac surgery: Experience in 148 patients” Mohr et al. 2001 reported on 17 patient undergoing robotic mitral valve surgery 14 of these patients had their procedure completed robotically. They concluded this type of robotic surgery was feasible and safe [114].

Typically surgical papers are published in a small number of specialised journals dedicated to that speciality [7]. In this analysis we have also found this to be the case. Despite searching 11 journals with a high impact factor and cardiac surgery theme our top 100 papers came from just 4 journals. Given the specialist nature of mitral surgery repair this is not entirely surprising. Papers discussing repair techniques are most often found in surgical themed journals. However, some papers discussing percutaneous valve repair and risk stratification can be found in Cardiology or medical journals with a high journal impact factor. In this analysis we have demonstrated that the inclusion of such journals is necessary to ensure high impact papers affecting mitral surgery are not overlooked.

Often highly cited papers are those that have been published less recently as they have time to accrue more citations. In our analysis this is not the case, with 37 of the top papers published after 2000; demonstrating the ongoing evolution of research in this field. The number of citations a paper receives is a limited measure of merit. However, it can be useful in judging the impact that paper has had on its field [10]. Journal impact factor is a better measure of merit in that a paper must be deemed of adequate quality to warrant publication in a widely read journal. Often this is determined by a small number of experts in that field. Peer Review is another mechanism that helps ensure papers published in journals with high impact factor are of high quality. Any research with flawed methodology is likely to be identified and a redaction made.

## Conclusion

Using this analysis, the most influential papers in mitral valve surgery in the modern era are those describing repair techniques. The most cited paper restored faith in mitral valve repair, improved its reliability and reduced the incidence of replacements. This often results in improved outcomes for patients not only from having their own mitral apparatus but avoiding prostheses and the need for lifelong anticoagulation.

Citation analyses of this type, while imperfect, do offer a useful overview of research in a field. Our bibliometric analysis demonstrates the progress made by mitral valve research and its future with investigation of percutaneous and robotic techniques. This analysis demonstrates the evidence-based approach used by cardiac surgeons today and the continued re-evaluation of mitral valve surgery to ensure the best possible outcomes for patients with mitral valve disease.

## Data Availability

The datasets used and/or analysed during the current study are available from the corresponding author on reasonable request.
